# Secondary care specialist visits made by children and young people prescribed antidepressants in primary care: a descriptive study using the QResearch database

**DOI:** 10.1186/s12916-020-01560-7

**Published:** 2020-04-30

**Authors:** Ruth H. Jack, Rebecca M. Joseph, Carol Coupland, Debbie Butler, Chris Hollis, Richard Morriss, Roger David Knaggs, Andrea Cipriani, Samuele Cortese, Julia Hippisley-Cox

**Affiliations:** 1 Division of Primary Care, School of Medicine, University of Nottingham, Nottingham, UK; 2 National Institute of Health Research MindTech MedTech Co-operative, The Institute of Mental Health, University of Nottingham, Nottingham, UK; 3 National Institute of Health Research Nottingham Biomedical Research Centre, Nottingham University Hospitals NHS Trust, Nottingham, UK; 4 Division of Psychiatry and Applied Psychology, School of Medicine, University of Nottingham, Nottingham, UK; 5 School of Pharmacy, University of Nottingham, Nottingham, UK; 6 Department of Psychiatry, University of Oxford, Oxford, UK; 7 Oxford Health NHS Foundation Trust, Warneford Hospital, Oxford, UK; 8 Center for Innovation in Mental Health, School of Psychology, Faculty of Environmental and Life Sciences, Clinical and Experimental Sciences (CNS and Psychiatry), Faculty of Medicine, University of Southampton, Southampton, UK; 9 Solent NHS Trust, Southampton, UK; 10 Department of Child and Adolescent Psychiatry, Hassenfeld Children’s Hospital at NYU Langone, New York, NY USA; 11 Nuffield Department of Primary Care Health Sciences, University of Oxford, Oxford, UK

**Keywords:** Antidepressants, SSRIs, Epidemiology, Primary health care, Child, Adolescent, Depression, Anxiety, Mental health

## Abstract

**Background:**

Antidepressants may be used to manage a number of conditions in children and young people including depression, anxiety, and obsessive-compulsive disorder. UK guidelines for the treatment of depression in children and young people recommend that antidepressants should only be initiated following assessment and diagnosis by a child and adolescent psychiatrist. The aim of this study was to summarise visits to mental health specialists and indications recorded around the time of antidepressant initiation in children and young people in UK primary care.

**Methods:**

The study used linked English primary care electronic health records and Hospital Episode Statistics secondary care data. The study included 5–17-year-olds first prescribed antidepressants between January 2006 and December 2017. Records of visits to paediatric or psychiatric specialists and potential indications (from a pre-specified list) were extracted. Events were counted if recorded less than 12 months before or 6 months after the first antidepressant prescription. Results were stratified by first antidepressant type (all, selective serotonin reuptake inhibitors (SSRIs), tricyclic and related antidepressants) and by age group (5–11 years, 12–17 years).

**Results:**

In total, 33,031 5–17-year-olds were included. Of these, 12,149 (37%) had a record of visiting a paediatrician or a psychiatric specialist in the specified time window. The majority of recorded visits (7154, 22%) were to paediatricians. Of those prescribed SSRIs, 5463/22,130 (25%) had a record of visiting a child and adolescent psychiatrist. Overall, 17,972 (54%) patients had a record of at least one of the pre-specified indications. Depression was the most frequently recorded indication (12,501, 38%), followed by anxiety (4155, 13%).

**Conclusions:**

The results suggest many children and young people are being prescribed antidepressants without the recommended involvement of a relevant specialist. These findings may justify both greater training for GPs in child and adolescent mental health and greater access to specialist care and non-pharmacological treatments. Further research is needed to explore factors that influence how and why GPs prescribe antidepressants to children and young people and the real-world practice barriers to adherence to clinical guidelines.

## Background

Antidepressant medicines are an important treatment option for a number of conditions in children and young people including depression [1], anxiety [2], and obsessive-compulsive disorder (OCD) [3]. In a 2017 survey, these conditions were found to affect 2.1% (depressive disorders), 7.2% (anxiety disorders), and 0.4% (OCD) of 5–19-year-olds in England [4]. In contrast, in 2015, the estimated prevalence of antidepressant use by 3–17-year-olds in the UK was 5 per 1000 patients, based on primary care data [5].

In the UK, the National Institute for Health and Care Excellence (NICE) produces guidelines for the treatment of many conditions. These guidelines contain evidence-based recommendations made by expert groups and are tailored to the UK health system. The guidelines also discuss the rationale behind recommendations. Regarding antidepressants, the NICE guideline for the treatment of depression in children and young people (NG134 [6]) states that prescribing of antidepressants to children and young people should follow assessment and diagnosis by a child and adolescent psychiatrist. The guidelines state fluoxetine should be prescribed in the first instance, and sertraline or citalopram considered if fluoxetine is unsuccessful or not tolerated.

Access to healthcare for both children and adults via the UK’s National Health Service (NHS) is centralised through general practitioners (GPs), who can initiate treatment and/or refer patients to specialists. The majority of people will therefore present to their GP in the first instance [7]. There is evidence that access to specialist Child and Adolescent Mental Health Services (CAMHS) is often difficult and can involve long waiting times (an average of 57 days for those both referred and treated in 2017/2018) [7, 8]. Faced with possible delays in accessing CAMHS, a GP might decide to initiate antidepressant treatment without prior specialist involvement. In studies based outside the UK, the level of specialist involvement in antidepressant prescribing varies. In France, where guidelines allow for non-specialist prescribing to adolescents [9], almost 75% of antidepressants prescribed to 6- to 17-year-olds in 2016 were initiated by GPs [10]. In Norway, on the other hand, 84.4% of antidepressant users aged 13 to 17 in 2012 had contact with specialist health care [11].

Despite their name, which was based on their first identified indication (depression) rather than their mechanism(s) of action [12], antidepressants are used to treat a number of conditions besides depression. In those aged under 18 years, antidepressants are licensed in the UK to treat depression, OCD, and nocturnal enuresis [13]. There is also evidence from systematic reviews and meta-analyses of the effectiveness of antidepressants in child and adolescent anxiety disorders [14, 15]. In studies of typical antidepressant prescribing to children and young people in the UK, depression was the predominant indication, followed by anxiety and pain [5, 16]. If the majority of antidepressants are prescribed to treat mental health conditions, then most antidepressant prescribing in this age group should be initiated by child and adolescent psychiatrists if NICE guidelines [6] are followed.

In this descriptive study, we used linked primary care and secondary care electronic health records to summarise the proportion of children and young people prescribed antidepressants in primary care who had also visited a relevant specialist. The second aim was to summarise the indications associated with these antidepressant prescriptions.

## Methods

The protocol for this study [17] contains full methodological details.

### Design and setting

This study is a descriptive observational cohort study utilising English primary care and linked secondary care electronic health records (EHR) between January 2006 and December 2017.

### Data source

The data source was QResearch (version 43), a database of anonymised routinely collected primary care EHR linked to secondary care inpatient and outpatient data (Hospital Episode Statistics (HES)). At the time of the study, the QResearch database included EHR for over 32 million patients from more than 1500 general practices across the UK. The EHR include patient characteristics, clinical diagnoses, symptoms, and prescription data. The primary care data are coded using a combination of Read Codes (version 2) and proprietary codes from EMIS Web, the computer system used by contributing practices.

The primary care EHR are linked to HES data on the basis of an anonymised version of the patient’s National Health Service (NHS) number, a unique identifier for patients across the UK. The inpatient and outpatient HES data contain information including episode dates, consultant specialty, and relevant diagnoses coded using the ICD-10 terminology.

### Cohort definition and study window

The study population was a subset of a larger cohort as described in the protocol [17]. Patients were eligible for inclusion if they were aged 5–17 years between 1 January 2006 and 31 December 2017 and were prescribed an antidepressant. This window follows the publication of the National Institute for Health and Care Excellence (NICE) guideline for depression in children and young people in 2005 [6]. Cohort entry was the latest date of the following: patient registration with their general practice plus 12 months, practice installation of the EMIS Web computer system plus 12 months, 1 January of the year the patient turned 5 years old, or 1 January 2006. Patients were followed up until the earliest date of the following: patient leaving the practice, patient death, 1 January of the year the patient turned 18 years old, or 31 May 2018. Patients were included if their first-ever antidepressant prescription was recorded during the study window. Patients were excluded if they had received an antidepressant prescription prior to cohort entry.

### Antidepressant prescriptions

Antidepressant prescriptions were identified in the primary care EHR using a pre-specified code list. The list was generated based on the associated chapter of the British National Formulary [18] (see protocol [17] for further details). Three categories were analysed: any antidepressant, selective serotonin reuptake inhibitors (SSRIs), and tricyclic and related antidepressants (TCAs). The ‘any antidepressant’ category includes all antidepressants, including those not categorised as an SSRI or TCA. The first-ever antidepressant prescribed within the study window was identified for each patient. Patients may be included in more than one category if they received a prescription for more than one type of antidepressant on the same day.

### Visits to specialists and antidepressant indications

Table 1 shows the pre-specified consultant specialities and antidepressant indications included. Information on secondary care consultant specialties was extracted from coded fields within the HES dataset. We extracted data for selected relevant specialties listed in the HES Data Dictionary [19], including ‘paediatrics’, ‘child and adolescent psychiatry’, and other psychiatry specialties. As per NICE guidelines for depression [6] and obsessive-compulsive disorder [20], initiation of antidepressants for mental health conditions in children and young people should be guided by a child and adolescent psychiatrist. It is possible some of the older members of the study population would interact with adult mental illness specialists, who treat those aged 18 years and over. For non-mental health conditions in children and young people in the UK, paediatricians provide specialist advice in the first instance.

**Table 1:** Antidepressant indications and consultant specialties examined

Indications	Consultant speciality
Anxiety	Adult mental illness
Attention deficit and hyperactivity disorder	Child and adolescent psychiatry
Autism	Forensic psychiatry
Depression	Paediatric neurology
Enuresis	Paediatrics
Neuropathic pain	Psychotherapy
Obsessive-compulsive disorder	
Phobias	
Self-harm	

Recorded antidepressant indications were obtained from the primary care data. For those under 18 years, antidepressants are licensed in the UK to treat depression, obsessive-compulsive disorder, and nocturnal enuresis. Although unlicensed in this age group, antidepressants may also be useful for treating anxiety disorders [14] and neuropathic pain [21]. Besides these indications, the following conditions were included as potential indications: attention deficit hyperactivity disorder (listed in the British National Formulary for Children as an unlicensed indication for imipramine [13], although not recommended in the guidelines [22]), phobias (a licensed indication in adults [18]), self-harm (closely associated with depression and anxiety [23]), and autism (evidence of increased prescribing [24] despite lack of evidence for efficacy [25, 26]). Clinical code lists (Read and EMIS codes) were used to find any records of the specified indications in the primary care data. Code lists were reviewed by members of the research team which included a practising general practitioner, mental health specialists, and epidemiologists with experience of UK EHR. The code list for depression included symptom codes as well as diagnosis codes, as there is some evidence that symptom codes are being increasingly used to code depression [16, 27]. We used a broad ‘enuresis’ code list to capture records of nocturnal enuresis.

Any records of the specified hospital visits or indications in the year before or 6 months after the first antidepressant prescription were identified. In the case of self-harm, the window was restricted to the year before the first antidepressant prescription so as not to include self-harm events occurring after a prescription. The categories were not mutually exclusive; thus, each patient may have more than one hospital visit/indication recorded.

### Patient characteristics

Sex (male/female) and age (based on the year of birth) were defined using the primary care EHR. Age was categorised as 5–11 or 12–17 years old at the time of the first antidepressant prescription. This was based on the age groups specified in the NICE guidelines [6] but excluded 18-year-olds, who may have been treated as adults. Ethnicity was classified according to the five broad ethnic groups used in the England and Wales 2001 Census: White, Mixed, Asian or Asian British, Black or Black British, and Chinese or other ethnic groups. Where a patient was missing ethnicity information in QResearch, the most recent valid ethnicity code in HES was used where available. Patients with no recorded ethnicity were included as a separate group. Townsend score [28] (at the patient postal code level) in quintiles was used as an indicator of socio-economic status. Patients were classified according to one of the nine regions in England to which their general practice belonged.

### Analysis

The baseline characteristics of those prescribed any antidepressant, SSRIs, and TCAs were summarised. The number and proportion of patients who had a record of visiting each of the secondary care specialities and the number and proportion of patients who had a record of the indications of interest were determined. These were summarised for the full study window (2006–2017) and for each individual year. The full result datasets for secondary care visits and indications over time are provided in Additional files 1 and 2. A minimum cell count of 5 (10 for combined groups) was used when presenting results in accordance with disclosure control guidance [29].

Data handling and analyses were performed using Stata/SE v15 (StataCorp LLC, TX) and R version 3.5.2.

### Patient and public involvement

The research team included a patient and public involvement representative (DB) who attended project meetings and guided the development of the protocol and interpretation of the results. DB is a member of the NIHR MindTech MedTech Involvement Team (https://www.mindtech.org.uk/involvement), a group of individuals with lived experience of mental health conditions. Discussion of the results with DB helped shape the final paper, with feedback incorporated into the ‘Discussion’ section.

### Ethics statement

The project has been reviewed in accordance with the QResearch agreement with East Midlands Derby Research Ethics Committee [reference 18/EM/0400].

## Results

In total, 33,031 children and young people met the study inclusion criteria. Of these, 2330 (7.1%) were aged 5–11 years and 30,701 (92.9%) were aged 12–17 years. The first antidepressant prescribed in primary care was an SSRI for 22,130 (67.0%) patients and a TCA for 10,489 (31.8%) patients. Of the SSRI prescriptions, 13,287 (60%) were for fluoxetine, 4641 (21%) were for sertraline, and 3945 (18%) were for citalopram. The characteristics of the study population are summarised in Table 2. The majority of patients were female (22,279, 67.4%), and 22,827 (69.1%) were classified as White (ethnicity information was missing for 24.0% of patients).

**Table 2:** Characteristics of the study population at the first antidepressant prescription, according to antidepressant class

	Any SSRI	Any TCA	Any antidepressant
*N*	22,130	10,489	33,031
Female, *n* (%)	14,960 (67.6%)	7054 (67.3%)	22,279 (67.4%)
Age at first prescription, median (IQR)	16 (15–17)	16 (13–17)	16 (15–17)
Aged 5–11, *n* (%)	652 (2.9%)	1659 (15.8%)	2330 (7.1%)
Aged 12–17, *n* (%)	21,478 (97.1%)	8830 (84.2%)	30,701 (92.9%)
Townsend quintile, *n* (%)			
1 (least deprived)	5481 (24.8%)	2670 (25.5%)	8234 (24.9%)
2	5554 (25.1%)	2515 (24.0%)	8156 (24.7%)
3	4891 (22.1%)	2200 (21.0%)	7195 (21.8%)
4	3912 (17.7%)	1798 (17.1%)	5788 (17.5%)
5 (most deprived)	2262 (10.2%)	1288 (12.3%)	3610 (10.9%)
Unknown	30 (0.1%)	18 (0.2%)	48 (0.2%)
Ethnicity, *n* (%)			
White	15,769 (71.3%)	6786 (64.7%)	22,827 (69.1%)
Mixed	298 (1.4%)	146 (1.4%)	451 (1.4%)
Asian or Asian British	541 (2.4%)	576 (5.5%)	1130 (3.4%)
Black or Black British	191 (0.9%)	219 (2.1%)	416 (1.3%)
Chinese or Other	177 (0.8%)	81 (0.8%)	265 (0.8%)
Unknown	5154 (23.3%)	2681 (25.6%)	7942 (24.0%)
Practice region, *n* (%)			
East Midlands	1092 (4.9%)	623 (5.9%)	1730 (5.2%)
East of England	1615 (7.3%)	869 (8.3%)	2510 (7.6%)
London	2387 (10.8%)	1341 (12.8%)	3780 (11.4%)
North East	824 (3.7%)	382 (3.6%)	1215 (3.7%)
North West	3915 (17.7%)	1499 (14.3%)	5498 (16.6%)
South East	6275 (28.4%)	2618 (25.0%)	9010 (27.3%)
South of England	2367 (10.7%)	1418 (13.5%)	3838 (11.6%)
West Midlands	2759 (12.5%)	1076 (10.3%)	3882 (11.8%)
Yorkshire and The Humber	896 (4.1%)	663 (6.3%)	1568 (4.8%)

*SSRI* selective serotonin reuptake inhibitor, *TCA* tricyclic and related antidepressant, *n* number, *IQR* interquartile range

**Fig. 1:**
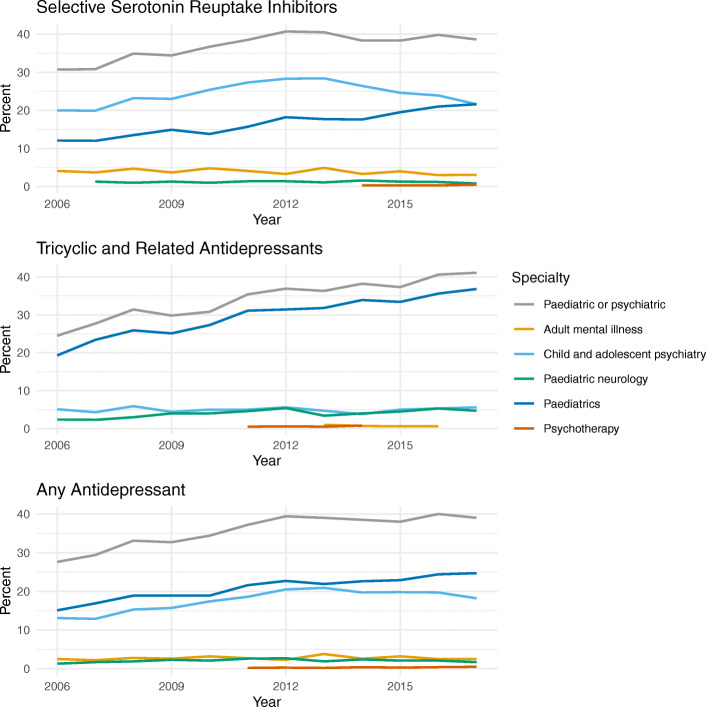
Percentage of the first antidepressant prescriptions associated with visits to hospital specialists over time. Includes visits made less than 1 year before or 6 months after the first antidepressant prescription. Specialties with fewer than 5 records have been masked

Overall, 12,149/33,031 (36.8%) children and young people in the cohort had a record of visiting a paediatric or psychiatric specialist in the 12 months before or 6 months after their first primary care antidepressant prescription. The figures were 8387/22,130 (37.9%) for SSRIs and 3625/10,489 (34.6%) for TCAs. Overall and for TCAs, the most common specialty recorded was paediatrics (7154/33,031 (21.7%) and 3144/10,489 (30.0%) respectively) (Table 3). Child and adolescent psychiatry was the specialty most frequently recorded in patients whose first antidepressant was an SSRI (5463/22,130, 24.7%). The proportion of those visiting a paediatric specialist increased steadily over the study period overall and in those prescribed SSRIs or TCAs. For those first prescribed SSRIs, the proportion of visiting a child and adolescent psychiatrist increased from 10.7% in 2006 to a peak of 19.3% in 2013 and then decreased to 16.9% in 2017 (Fig. 1).

**Table 3:** Visits to hospital specialists associated with the first antidepressant prescription

Consultant specialty	Ages 5–11 (*n* = 2330)	Ages 12–17 (*n* = 30,701)	SSRI (*n* = 22,130)	TCA (*n* = 10,489)	Any antidepressant ages 5–17 (*n* = 33,031)
Any paediatric or psychiatric	1157 (49.7%)	10,992 (35.8%)	8387 (37.9%)	3625 (34.6%)	12,149 (36.8%)
Adult mental illness	13 (0.6%)	900 (2.9%)	833 (3.8%)	54 (0.5%)	913 (2.8%)
Child and adolescent psychiatry	285 (12.2%)	5770 (18.8%)	5463 (24.7%)	522 (5.0%)	6055 (18.3%)
Forensic psychiatry	< 10	10 (< 0.1%)	< 10	< 10	10 (< 0.1%)
Paediatric neurology	124 (5.3%)	567 (1.8%)	259 (1.2%)	424 (4.0%)	691 (2.1%)
Paediatrics	1000 (42.9%)	6154 (20.0%)	3940 (17.8%)	3144 (30.0%)	7154 (21.7%)
Psychotherapy	13 (0.6%)	79 (0.3%)	53 (0.2%)	39 (0.4%)	92 (0.3%)

Includes visits made less than 1 year before or 6 months after the first antidepressant prescription. Individuals may be included in both SSRI and TCA categories and may have visited more than one type of specialist. Counts less than 10 have been masked

*SSRI* selective serotonin reuptake inhibitor, *TCA* tricyclic and related antidepressant, *n* number

**Fig. 2:**
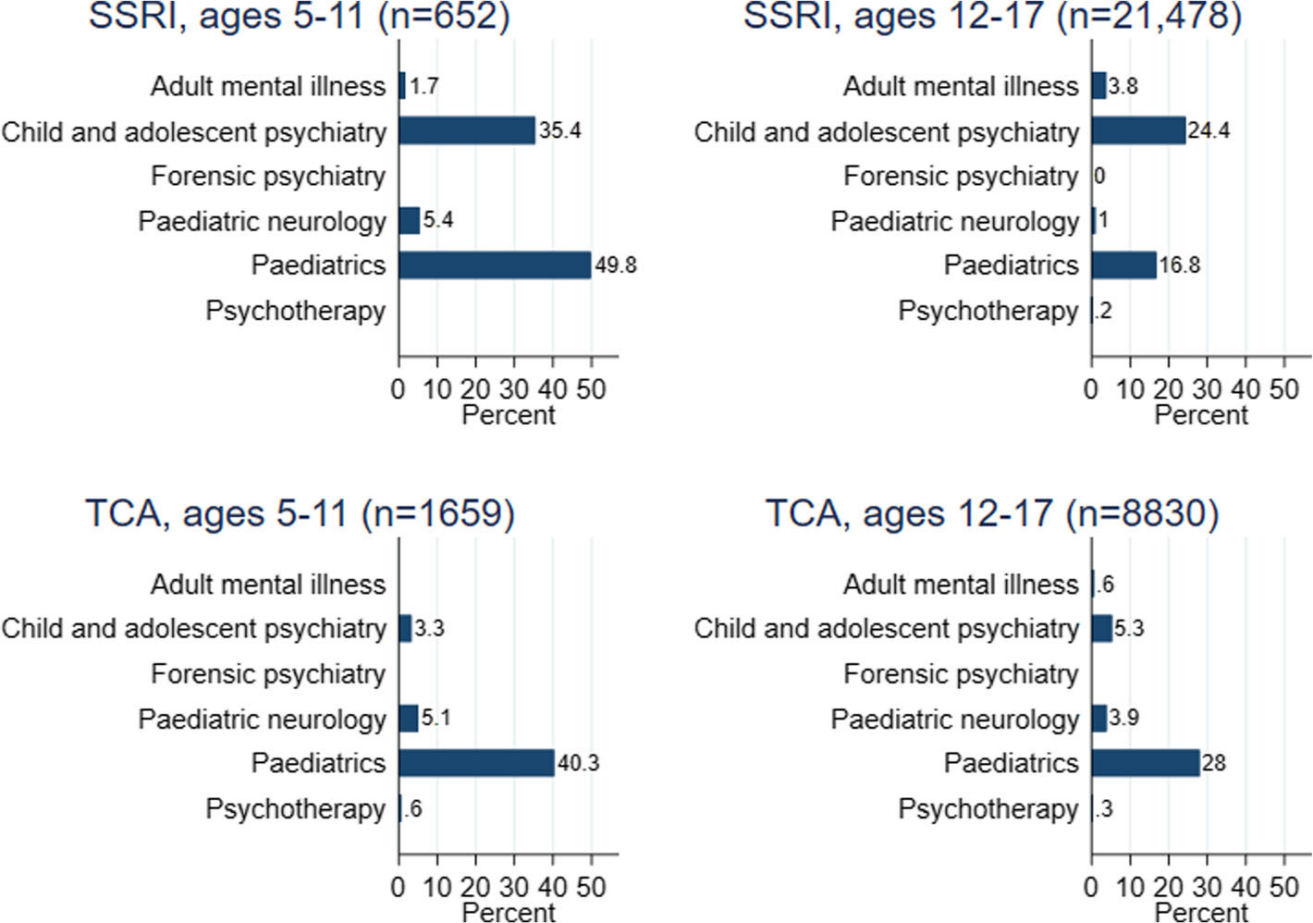
Percentage of the first antidepressant prescriptions associated with visiting a hospital specialist. Includes visits made less than 1 year before or 6 months after the first antidepressant prescription. SSRI, selective serotonin reuptake inhibitor; TCA, tricyclic and related antidepressant; *n*, number. Specialties with fewer than 5 records have been masked

By patient age, 1157/2239 (49.7%) 5–11-year-olds and 10,992/30,701 (35.8%) 12–17-year-olds had a record of visiting a paediatric or psychiatric specialist (Table 3). Visits to specialists according to patient age and antidepressant type are summarised in Fig. 2. In both age groups, those prescribed TCAs were more likely to have visited a paediatric than a psychiatric specialist. For SSRIs, 5232 /21,478 (24.4%) 12–17-year-olds had visited a child and adolescent psychiatrist.

**Fig. 3:**
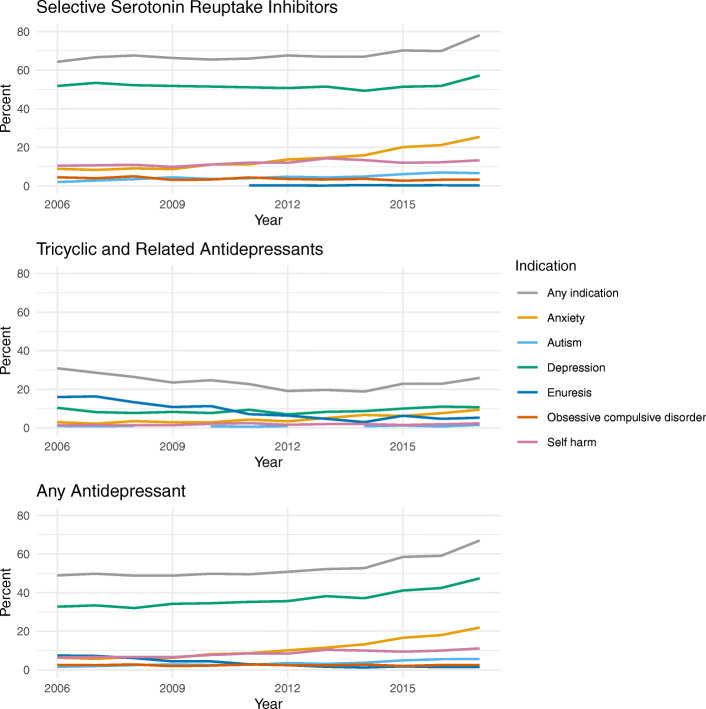
Percentage of the first antidepressant prescriptions associated with specified indications over time. Includes indications recorded less than 1 year before or 6 months after the first antidepressant prescription. Indications with fewer than 5 records have been masked. Attention deficit hyperactivity disorder, phobias, and neuropathic pain are not shown to improve readability (all had consistently low percentages)

Overall, 17,972/33,031 (54.4%) children and young people in the cohort had a record of at least one of the indications of interest in the 12 months before or 6 months after their first primary care antidepressant prescription. This increased over time from 48.9% in 2006 to 67.0% in 2017 (Fig. 3). The most frequently recorded indications were depression (*n* = 12,630, 38.2%) and anxiety (*n* = 4155, 12.6%) (Table 4). For SSRIs, 15,295/22,130 (69.1%) first prescriptions were assigned an indication, and for TCAs, the proportion was 2463/10,489 (23.5%). The most frequently recorded indication for both SSRIs (11,537/22,130, 52.1%) and TCAs (936/10,489, 8.9%) was depression (Table 4). Considering the other licensed indications, 774/22,130 (3.5%) SSRI prescriptions had an indication of obsessive-compulsive disorder recorded and 877/10,489 (8.4%) TCA prescriptions had a record of enuresis. Over the study window, the proportion of TCA prescriptions associated with enuresis decreased from 16.0 to 5.3%. The proportion of all first antidepressant prescriptions with records of anxiety or depression increased between 2006 and 2017 from 6.3 to 21.9% for anxiety and 32.7 to 47.4% for depression (Fig. 3).

**Table 4:** Indications associated with the first antidepressant prescription

Indication	Ages 5–11 (*n* = 2330)	Ages 12–17 (*n* = 30,701)	SSRI (*n* = 22,130)	TCA (*n* = 10,489)	Any antidepressant ages 5–17 (*n* = 33,031)
Any of the pre-specified indications	998 (42.8%)	16,974 (55.3%)	15,295 (69.1%)	2463 (23.5%)	17,972 (54.4%)
Anxiety	136 (5.8%)	4019 (13.1%)	3601 (16.3%)	508 (4.8%)	4155 (12.6%)
Attention deficit hyperactivity disorder	< 10	61 (0.2%)	50 (0.2%)	14 (0.1%)	66 (0.2%)
Autism	206 (8.8%)	1020 (3.3%)	1127 (5.1%)	90 (0.9%)	1226 (3.7%)
Depression	72 (3.1%)	12,558 (40.9%)	11,537 (52.1%)	936 (8.9%)	12,630 (38.2%)
Enuresis	642 (27.6%)	308 (1.0%)	73 (0.3%)	877 (8.4%)	950 (2.9%)
Neuropathic pain	< 10	104 (0.3%)	< 10	107 (1.0%)	113 (0.3%)
Obsessive compulsive disorder	53 (2.3%)	751 (2.4%)	774 (3.5%)	28 (0.3%)	804 (2.4%)
Phobias	< 10	260 (0.8%)	222 (1%)	40 (0.4%)	268 (0.8%)
Self-harm	10 (0.4%)	2948 (9.6%)	2715 (12.3%)	190 (1.8%)	2958 (9.0%)

Includes indications recorded less than 1 year before or 6 months after the first antidepressant prescription. Individuals may be included in both SSRI and TCA categories and may have records of more than one indication. Counts less than 10 have been masked

*SSRI* selective serotonin reuptake inhibitor, *TCA* tricyclic and related antidepressant, *n* number

**Fig. 4:**
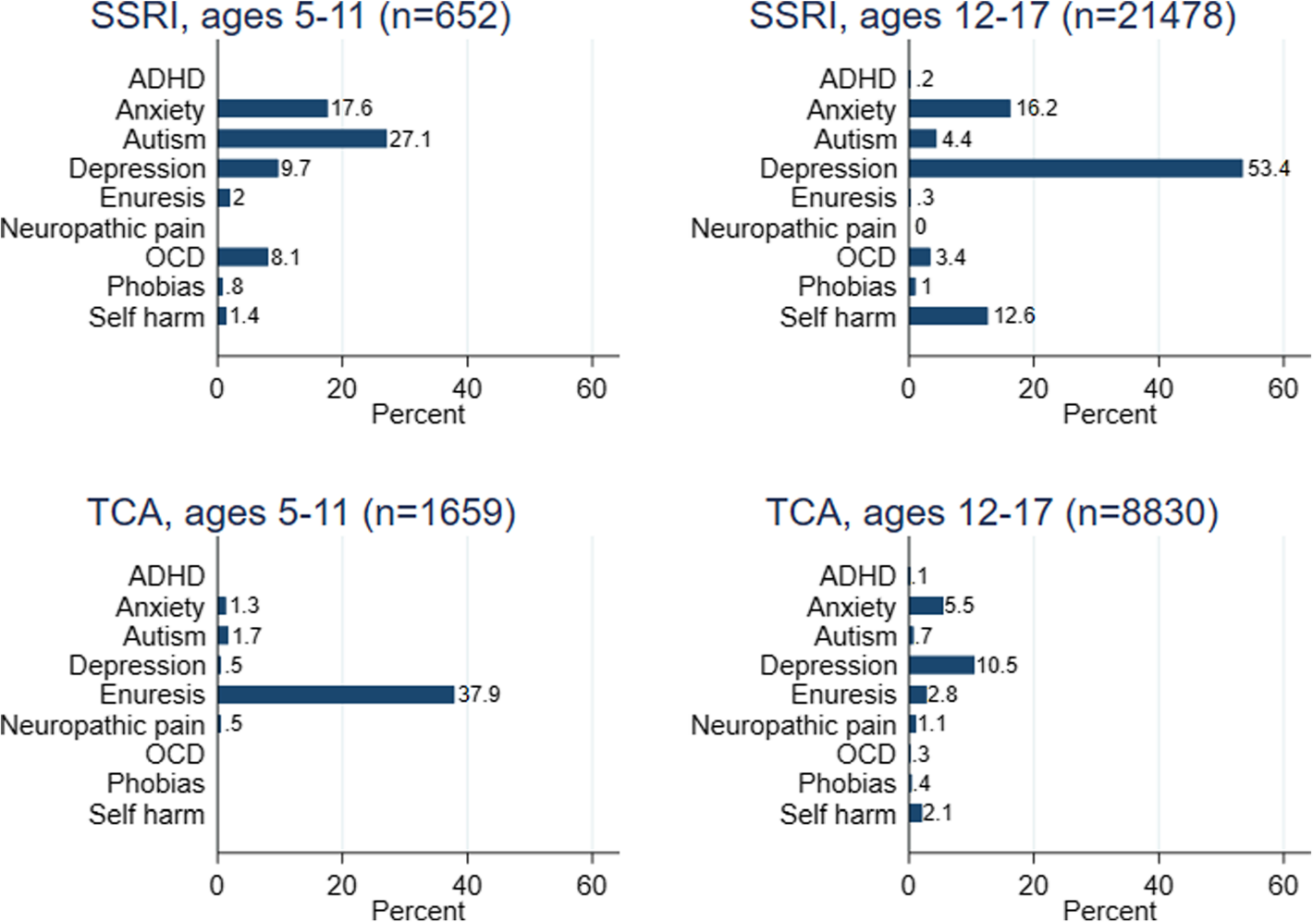
Percentage of the first antidepressant indications associated with each recorded indication. Includes indications recorded less than 1 year before or 6 months after the first antidepressant prescription. SSRI, selective serotonin reuptake inhibitor; TCA, tricyclic and related antidepressant; *n*, number; OCD, obsessive-compulsive disorder; ADHD, attention deficit hyperactivity disorder. Indications with fewer than 5 records have been masked

For those aged 5–11 years, 998/2330 (42.8%) had a recorded indication, and the most frequently recorded indications were enuresis (642/2330, 27.6%) and autism (206/2330, 8.8%) (Table 4). For those aged 12–17 years, 16,974/30,701 (55.3%) had a recorded indication, and the most frequently recorded indications were depression (12,558/30,701, 40.9%) and anxiety (4019/30,701, 13.1%). Indications according to age and antidepressant type are summarised in Fig. 4. Over half of the 12–17-year-olds first prescribed SSRIs had a record of depression (11,474/21,478, 53.4%).

## Discussion

This study included 33,031 children and young people in England who were prescribed an antidepressant in primary care between January 2006 and December 2017. Of these, 12,149 (37%) had a record of visiting a secondary care paediatric or psychiatric specialist less than 12 months before or 6 months after their first primary care antidepressant prescription. The percentage of 5–17-year-olds prescribed antidepressants with a record of visiting a specialist increased between 2006 and 2012, before levelling off at approximately 39%. Considering only those aged 12–17 years old prescribed SSRIs, 24.4% had a record of visiting a child and adolescent psychiatrist. Although the proportion of visiting a child and adolescent psychiatrist initially increased, it began to decrease after 2013.

These results suggest a significant proportion of children and young people receive prescriptions for antidepressants without the involvement of a relevant specialist, such as a mental health specialist or paediatrician. This is contrary to the NICE guidelines for depression [6] and obsessive-compulsive disorder [20], which recommend assessment and diagnosis by a child and adolescent psychiatrist before the initiation of antidepressants, and nocturnal enuresis [30], which recommends referral for further review and assessment of possible underlying factors before initiation of imipramine. There could be several reasons for this, including the lack of awareness of relevant guidelines by GPs. However, one small qualitative study suggested good understanding of the depression guidelines amongst GPs in interviews [31]. Another explanation for GPs initiating antidepressants without the involvement of a specialist could be a lack of prompt access to secondary care services or evidence-based non-pharmacological treatments such as cognitive behavioural therapy. There is limited availability of specialist services leading to long waiting times (average 57 days in 2017/2018) [8] and strict acceptance criteria [32]. According to reports by the Children’s Commissioner for England, provision of low-level (non-specialist, preventative, and early intervention) mental health services is complicated and variable across the country [33]. If a GP feels a child or young person needs urgent treatment for their mental health condition, and other non-pharmacological options and specialist assessment are not immediately available, they may choose to prescribe antidepressants despite the recommendations of clinical guidelines. In addition, patients or their parents may decline other treatment options or request antidepressants. For example, some of the adolescents in one qualitative study described that without SSRIs, they felt too low to benefit from alternative therapies [34].

We also explored the indications associated with antidepressant prescribing to children and young people. Where indications were identified, depression was the most frequently recorded indication overall (38%), echoing the findings of other studies utilising UK EHR [5, 16, 35]. John et al. [16] found over half of new antidepressants prescribed to 6–18-year-olds between 2003 and 2013 were associated with a depressive disorder or depression-related symptoms. Like this earlier study, we used a broad code list to capture depression which included symptom codes (e.g. ‘low mood’) as well as diagnostic codes. The figure we report is therefore likely to be higher than the true proportion of patients with a diagnosis of depression. Furthermore, the depression code list included mixed anxiety and depression codes (not included in the anxiety code list). Depression and anxiety often co-exist, particularly in older children and adolescents.

We were unable to identify an indication for a large proportion of patients in the cohort (46% overall). This was the case for around a third of patients first prescribed SSRIs (31%) and much higher for those first prescribed TCAs (77%). The proportion of patients with a record of one of the indications of interest increased over the study window, particularly for those prescribed SSRIs. For patients prescribed SSRIs in the final year of the study period (2017), 57% had a record of depression and 25% had a record of anxiety. In this study, we used a pre-specified list of indications, and it is possible that some indications were missed. Sarginson et al. explored antidepressant prescriptions to 3–17-year-olds between 2000 and 2015 using UK EHR [5]. They reported a number of indications in addition to those in our study, but all were associated with a small percentage of prescriptions: eating disorder (0.8%), headache disorder (2.3%), and abdominal pain/irritable bowel syndrome (2.3%) [5].

Overall, missing information about antidepressant indications makes it difficult to assess whether antidepressants were prescribed for evidence-based indications (whether licensed or ‘off-label’). Despite this, it may be reasonable to assume the majority of SSRIs were prescribed to treat mental health conditions, particularly towards the end of the study period. As discussed later, several factors may influence clinical coding. The use of codes in UK primary care is generally at the clinician’s discretion, and the lack of a coded indication does not necessarily imply a gap in care provision.

Strengths of this study include the use of QResearch, a large, population-based primary care database which captures all prescriptions issued from participating general practices, and linkage with a secondary care database which captures all NHS hospital visits made by patients in England. The QResearch database includes data from a sample of practices using EMIS Web software, the most widely used and widely distributed clinical computer system in England [36]. As a result, the study cohort is likely to be a representable sample of children and young people in England, and the study findings are likely to be generalisable to primary care across the country. Another strength is that our time window for specialist visits includes the immediate period following as well as before primary care initiation of prescribing. This allows for the possibility that in urgent cases, prescribing may be initiated in primary care together with a secondary care referral. Hence, we are unlikely to have underestimated the true proportion of antidepressant prescribing where there has been no secondary care involvement.

The following limitations should be considered when interpreting the results of this study. First, it is possible that we did not capture all interactions with specialists. While the majority of specialist consultants in paediatrics and CAMHS are likely to be based in secondary care, it is possible some may be based in a community setting which would not be captured within HES. The HES dataset does not include data about privately funded medical care. However, in the UK, the vast majority of children and young people receive healthcare provided by the NHS. This study will not have captured any hospital visits that occurred more than 12 months before or 6 months after the first primary care prescription was issued. Misclassification within the consultant specialty field of the HES dataset could also have resulted in some visits being missed. However, in this study, the association between antidepressant prescriptions and specialist visits was based entirely on their relative timing, and many of the visits (particularly to paediatrics) may have been for unrelated issues. On balance, therefore, we believe these figures are more likely to be overestimates.

Second, we identified indications based on the diagnostic codes recorded by clinicians. Indications would only be captured if coded in the patient’s record and if the code lists we applied captured all relevant cases. UK general practice records contain a mixture of coded and free-text information, and coding can depend on factors such as severity, whether the consultation is the first presentation of symptoms, and whether the condition is included in the Quality and Outcomes Framework (QOF) which provides financial incentives for the management of certain conditions [37]. In this study, it is possible that conditions were recorded in free-text records but never coded, and thus unavailable in the datasets used. Originally, we planned to supplement the primary care data with diagnosis information from HES. Upon examination, however, the secondary care data identified very few additional cases (e.g. 3% increase in the number of patients with a depression diagnosis code), and so only the primary care data were used for this purpose. The code lists used in this study were created by experienced clinicians with expertise in UK primary care and psychiatry. The majority of code lists (except depression, as discussed earlier) included diagnostic codes but not symptom codes. It is possible that the frequency of indications with these narrower definitions was underestimated, depending on the coding preferences of clinicians. In summary, it is possible that relevant information about antidepressant indications is available to clinicians while providing clinical care. However, based on the information typically available at scale to researchers, it is difficult to identify antidepressant indications for a significant proportion of the population of interest.

Third, the prescription data only represent prescriptions issued within primary care. The prescriptions included in the study may not be the patients’ first-ever antidepressant if the first prescription was issued in secondary care. However, when the first prescription occurs in secondary care, prescribing will normally be transferred to primary care, meaning these patients are likely to be represented in our analysis. The full clinical picture will be more complex than presented in this study and conclusions about prescribing behaviour should be limited to primary care.

This paper does not provide any new evidence about the rates of antidepressant prescribing in this population or possible changes to the relative frequency of prescribing of different antidepressants. Several existing studies have demonstrated increases in antidepressant prescribing in the years up to 2015 [5, 16, 35, 38, 39], and in future work, we will explore whether these trends have continued in more recent years (see protocol [17]).

## Conclusions

A minority (37%) of children and young people prescribed antidepressants in English primary care had a record of visiting an NHS-funded paediatric or psychiatric specialist less than 12 months before or 6 months after their first antidepressant prescription. While a small proportion of patients may have visited specialists outside the NHS, overall, these results suggest that many children and young people are being prescribed antidepressants without the involvement of a relevant specialist. This practice diverges from UK clinical guidance and may represent difficulty accessing specialists or evidence-based non-pharmacological interventions. These findings may justify both greater training for GPs in child and adolescent mental health and greater access to specialist care and non-pharmacological treatments. In 46% of patients, it was not possible to determine the likely indication for the antidepressants; thus, it is difficult to assess whether the antidepressants were prescribed for evidence-based indications. Further research is needed to explore the factors that influence how and why GPs prescribe antidepressants to children and young people and the real-world practice barriers to adherence to clinical guidelines.

## Supplementary information

**Additional file 1.** VisitsOverTime.csv, data showing percentage of patients with a visit to each specialty by year and antidepressant type (used for Fig. 1).

**Additional file 2.** IndicationsOverTime.csv, data showing percentage patients with a record of each indication by year and antidepressant type (used for Fig. 3).

## Data Availability

The datasets analysed during the current study are not publicly available as they are provided under agreement with QResearch (https://www.qresearch.org/). The patient-level data from the QResearch database are specifically licensed according to its governance framework, see http://www.qresearch.org for details. The full statistical code is available from the authors.

## Abbreviations

CAMHS: Child and Adolescent Mental Health Services
EHR: Electronic health records
EMIS: Egton Medical Information Systems (a supplier of electronic patient record systems)
GP: General practitioner
HES: Hospital Episode Statistics
NHS: National Health Service
NICE: National Institute for Health and Care Excellence
NIHR: National Institute for Health Research
QOF: Quality and Outcomes Framework
SSRI: Selective serotonin reuptake inhibitor
TCA: Tricyclic and related antidepressant
UK: United Kingdom
